# Monitoring and evaluation of childhood stunting reduction program based on fish supplement product in North Sumatera, Indonesia

**DOI:** 10.1038/s41598-024-61462-z

**Published:** 2024-05-22

**Authors:** Bens Pardamean, Rudi Nirwantono, Alam Ahmad Hidayat, Joko Pebrianto Trinugroho, Mahmud Isnan, Reza Rahutomo, Digdo Sudigyo, Faisal Asadi, Gregorius Natanael Elwireharja, Dedy Ariansyah, Ratna Sari, Roma Dame Uli Pasaribu, Guntur Berlian, Muhammad Ichwan, Sarma Nursani Lumbanraja

**Affiliations:** 1 Bioinformatics and Data Science Research Center, Bina Nusantara University, Jakarta, Indonesia; 2 Department of Health of Serdang Bedagai Regency, Sei Rampah, Indonesia; 3 School of Pharmacy, Bandung Institute of Technology, Bandung, Indonesia; 4 Department of Pharmacology and Therapeutic, Faculty of Medicine, Universitas Sumatera Utara, Medan, Indonesia; 5 Department of Obstetic and Gynecology, Faculty of Medicine, Universitas Sumatera Utara, Medan, Indonesia

**Keywords:** Nutritional supplements, Health policy, Health services, Nutrition, Paediatrics, Public health, Epidemiology, Outcomes research

## Abstract

The government of Serdang Bedagai Regency initiated a supplementation program to reduce the high prevalence of stunting in the area by delivering extra supplementation, which were nutritious biscuits from national government and fish-based supplement produced from local resources. A 6-month study from April 2022 to September 2022 was conducted to monitor and evaluate the government program that involved 219 under-5-year-old children with height-for-age Z-score (HAZ-score) below − 2. We observed the stunting prevalence reduction by 37.00%, where 81 children recovered from stunting (HAZ-score ≥ − 2). Furthermore, the mean HAZ-score and WHZ-score (Weight-for-Height Z-score) were monitored to significantly improve by 0.97 ± 1.45 (*P*-value = 1.74e^−14^) and 1.00 ± 2.18 (*P*-value  = and 2.40e^−8^), subsequently. The most significant improvement in HAZ-score was monitored among children receiving fish-based supplements with 1.04 ± 1.44 improvement (*P*-value = 6.59e^−17^). Then, a significant WHZ-score improvement was reported from children consuming fish-based supplements and a combination of fish-based supplements with nutritious biscuits (*P*-value = 2.32e^−8^ and 5.48e^−5^) by 1.04 ± 2.29 and 0.83 ± 1.84, respectively. The results of the observation become evidence that the program could effectively reduce the prevalence of stunting in children below five years old, especially among children who received locally produced fish-based supplements.

## Introduction

Currently, stunting remains a significant health burden in developing countries. The World Health Organization (WHO) reported that the global prevalence of stunting was nearly the same percentage in the Asia–Pacific region, amounting to 22.0% in 2020^1,2^. In 2021, the prevalence of stunting in Indonesia was approximately 24.4%, with almost one-quarter of the affected individuals being children under five years-old^3^.

The Ministry of Health of Indonesia has implemented a nationwide program to eliminate stunting. This endeavor encompasses the provision of supplementary nutrition in the form of biscuits enriched with essential nutrients. The Supplementary Feeding Program (SFP), which offers biscuits for Indonesian children, is implemented to address nutritional disorders during their growth period. The biscuits are administered periodically to children aged 6 to 59 months as participants in the programs. The participants were provided with twelve pieces of biscuits or three packs per day a week, and their nutritional status was monitored by the local community health care center or local health officers. The supplementary has been approved for nationwide implementation and each implementation will last for a duration of 90 consecutive days^4^.

A stunting reduction program is also intensively conducted in a newly established regency, Serdang Bedagai Regency in North Sumatera Province, Indonesia, by the local government of the regency due to the high prevalence of stunting in the area. The program is one of the priority programs under regent command due to the high prevalence of stunting in the region with up to 30.8% in 2018 and raised to 36,2% in 2019 or higher than the national average. However, the prevalence reduced to 24,4 percent in 2021 and 21.1% in 2022^5^. Therefore, the Health Department of Serdang Bedagai Regency, along with the National Population and Family Planning Board and the Empowerment of Family Welfare Association, work together to monitor the case, give appropriate treatments, and provide direct supervision to the families with stunting children.

The program involves the delivery of biscuits that are provided by the national government (Supplementary Feeding Program) and fish-based supplements that are locally produced. The fish-based supplement delivery proposed by the local government accompanies the biscuits delivery due to the limitation of biscuits stock provided by the national government. Besides, the fish-based supplement was introduced by the local government since the stripped snakehead fish (*Channa striata*) is abundant in Serdang Bedagai Regency. It also becomes a viable option due to its affordability and acceptability in taste among the general population. The fish-supplement in the form of liquid fish extract was then combined with two herbal appetite stimulants, e.g., *Curcuma xanthorrhiza* and *Phyllanthus niruri*. Thus, it is the best option as a source of local food to support the children’s supplementation.

However, the government of Serdang Bedagai has no adequate instrument to monitor the program intensively and evaluate the progress of the provision of specialized nutrition supplementation. On other hand, conducting an intensive monitoring and proper evaluation during the stunting reduction program could improve the success of the program. Besides, the result of observation can be a suggestion for the government to improve the program and allocate the budget efficiently. Therefore, this study is established to support the local government of Serdang Bedagai in monitoring the program and observing the progression and successful of the supplementary program according to children’s anthropometric parameters. We registered the children with stunting in Serdang Bedagai Regency and observed the progression during the stunting supplementary program conducted by the local government. The data collection was performed using a database application, StuntingDB, which was developed by the Bioinformatics and Data Science Research Center (BDSRC) of Bina Nusantara University. The data were updated by midwives, nutritionists, and pediatric associates from direct measurement.

## Material and methods

### Research site and local government program

**Figure 1:**
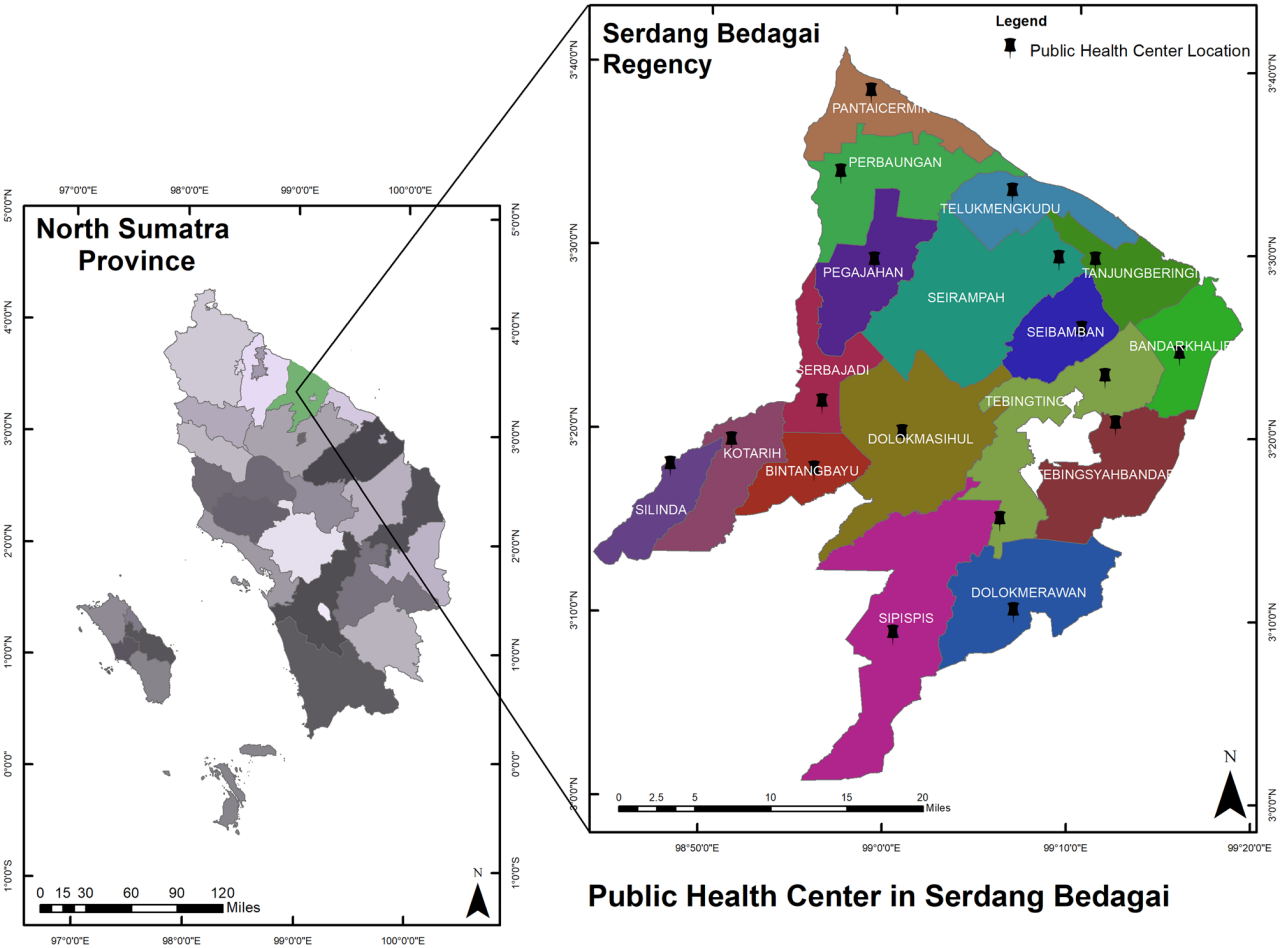
Map of Serdang Bedagai Regency and the location of public health centers.

The data were collected from 17 public health center locations in Serdang Bedagai Regency, with an area of 1,900.22 km^2^, in North Sumatera Province, Indonesia. Figure 1 shows the map of the public health center in Serdang Bedagai generated with ArcGIS 10.2 (freely used software for a publication purpose)^6^. Data from 2019 from the BPS—Statistics Indonesia showed that the total population in Serdang Bedagai Regency was 616,396 inhabitants, with a total of male 50.19%, female 49.81%, and the ratio with a sex ratio of 100.77^7^. In addition, the data from the Indonesia Ministry of Health based on the Results of the Indonesian Nutritional Status Survey in the year 2022^8^ showed the prevalence of stunting data in the Serdang Bedagai regency was 21.1%, which was similar to the prevalence in the North Sumatra province.

The government conducted a supplementary program in the area to reduce the percentage of stunting cases. The program was performed by delivering nutritious biscuits from the National Supplementary Feeding Program (SFP) and fish-based supplement as the local food. The fish-based supplement was distributed by the local government. In the supplement preparation, the local government collaborated with Akar Rimba Nusantara and Mega Medica Pharmaceuticals, local pharmaceuticals companies based in the area. The fish-supplement was in the form of liquid extract of stripped snakehead fish (*Channa striata*) that was combined with two herbal appetite stimulants, e.g., *Curcuma xanthorrhiza* and *Phyllanthus niruri*. The supplements were delivered to the children and consumed daily.

### Participants and data collection

The observation was conducted in 6 months from April 2022 to September 2022 with the participation of potentially age-eligible children from 20 regency-level public health centers in the research site. The eligible participants were initially identified by the midwives. The children included in this study were those who met following the criteria: children under 55 months, reside in the studied area, and signed the informed consent from a legal guardian. The data collected in this study were demographics, birth dates, and anthropometric measurements of the children. Prior to the data imputation to the application, we also monitored the accuracy of the midwives during the anthropometric measurement and performed standardization. The data collected was then inputted into performed using the StuntingDB application by pre-trained midwifes as the application users.

The StuntingDB application is a database management system (DBMS) that was designed and developed by Bioinformatics and Data Science Research Center (BDSRC) in 2020 to manage stunted children’s data. The StuntingDB was designed to connect stunted children’s profile and their personal anthropometric measurements to gain reliable data and information in childhood stunting research by using personal computers, tablet computers, or smartphones^9,10^. StuntingDB replaced the traditional way of recording anthropometric measurements which relies on logbooks with digital data recording in order to provide a systematic workflow and reduce the level of human error^11^. For the most part, users are provided with the ability to manage child growth data and access child growth reports. Specifically, users are allowed to track changes in individual anthropometric measurements over a period of time.

Working with StuntingDB started with user registrations. Users then register participants by completing a survey form and initial measurement including height, weight, frequency of fever, diarrhea, and appetite in a month. Users inserted child anthropometric measurements once every month for six months. The activity was followed by data curation to ensure the inserted data was valid and accountable. There are several cases where participants were excluded from the research, such as moving out of the research site, passing away, or declaring to quit the research. After the data collection period, the research was continued with data extraction and analysis.

### Data filtering

**Figure 2:**
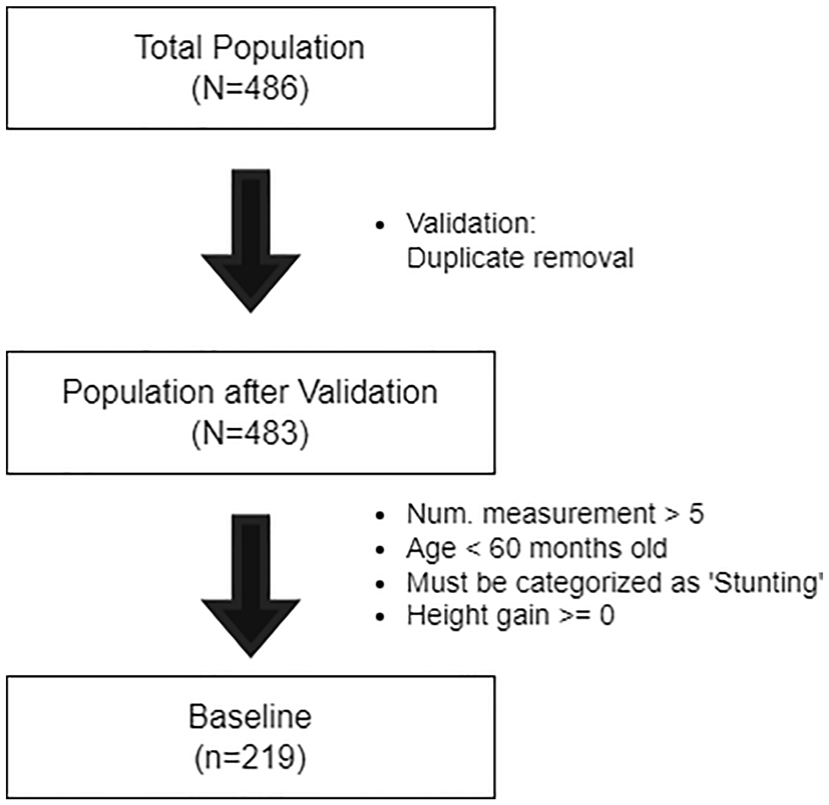
The steps in data filtering.

Following the data collection, two data validation were applied for data pre-processing (Fig. 2). The first data validation focused on duplicate removal. It produced an overview of the children population (N = 483). Following the first validation, the second validation was performed to filter the data by applying five exclusion criteria, which were the child must be categorized as stunting in the initial observation, receive supplementation, the number of measurements must be more than five, age must be less than 60 months old by the end of data collection period, height gain must be more than or equal to 0. The stunting criteria were defined by standard anthropometrics from the WHO Child Growth Standard. The height-for-age z-score (HAZ) was utilized as the standard anthropometric measurement to categorize the stunted status of the participants in the program. The participants with the measured range of − 3 < HAZ < − 2 were considered to be stunted, while the severely stunted participants were HAZ < − 3. In this program, only participants with HAZ < − 2 (stunted or severely stunted) underwent further analysis since we wanted to focus on monitoring the progression of the supplementation program conducted by the government. In addition, a complementary variable weight-for-height z-score (WHZ) was also used to determine the wasted status. Defining the moderately wasted status as − 3 < WHZ < − 2 and the severely wasted participants as WHZ < − 3.

### Data analysis

Anthropometric indexes including height-for-age z-score (HAZ), weight-for-age z-score (WAZ), and weight-for-height z-score (WHZ) were calculated before and after supplementation using the WHO Anthro Software Nutrition Survey module called the Anthro Survey Analyzer, which conforms to the WHO Child Growth Standard. Statistical analysis was then conducted using participant t-tests to compare mean values of continuous variables collected in the initial data observation and after six months of observation. If the data did not follow a normal distribution, Mann–Whitney U test was employed. One-Way ANOVA test was utilized to assess the null hypothesis that there is no significant difference between the cohorts. Tukey–Kramer tests were additionally conducted to determine pairwise associations within groups when the ANOVA test produced a statistically significant result.

### Ethics declarations

The ethical review was approved by the Health Research Ethical Committee, School of Medicine, North Sumatera University (Universitas Sumatera Utara/USU) in North Sumatra Province-Indonesia, based on ethical clearance number 1016/KEPK/USU/2022. The datasets analysed in current research are not publicly available since the study was conducted in a collaboration with the local government of Serdang Bedagai Regency and we share the data ownership with them. However, the data can be available from the corresponding author on reasonable request.

## Results

Table 1 presents the characteristics of the children population (n = 219) who were eligible in this study after exclusion. After this data cleansing, the validated participants were known to be assigned to 17 public health centers. The average age was 29.66 ± 11.88 months old. The number of male and female children were 107 (48.86%) and 112 (51.14%), respectively. The average height was 79.06 ± 8.38 cm, and the average weight was 9.50 ± 2.23 kg.

**Table 1:** Characteristics of Children Population from the First Data-Validation.

Demographic characteristics	Count
Mean age [month]	29.66 ± 11.88
Sex	
Male	107 (48.86%)
Female	112 (51.14%)
Mean height [cm]	79.06 ± 8.38
Mean weight [kg]	9.50 ± 2.23

The total percentage of recruited participants with moderately stunted status in the initial observation was 54.79% (n = 120), and severely stunted was 45.21% (n = 99). Along with this, the wasting children observed in this study were 29 children (13.24%), severely wasting children were 33 children (15.07%), and the rest were normal (71.69%). The overall mean HAZ score was − 3.26 ± 1.06, and the overall mean WHZ score was − 0.90 ± 1.74.

Further, the observation revealed that during the supplementary program by the government of Serdang Bedagai Regency, the children were separated into three different categories based on the supplement they received: fish-based supplement, children supplemented with nutritious biscuits from National Supplementary Feeding Program (nutritious biscuit), and combination both of them (Table 2). However, the number of children in each category was obviously imbalance. The children who received only fish-based supplement was the most abundant population (n = 166 children), followed by children who received combination supplement (n = 39). While the children received nutritious biscuits only was the less abundance among all categories (n = 14). Nevertheless, mean HAZ score of all categories of supplementation were almost similar, which were − 3.20 ± 1.01, − 3.62 ± 1.00, and − 3.28 ± 1.20 for fish-based supplement, nutritious biscuits, and combination, respectively. The group that received fish-based supplementation was also monitored to own the least mean WHZ score of − 0.92 ± 1.76, while those who were in nutritious biscuits and combination recorded mean WHZ scores of − 1.69 ± 1.38 and − 1.38 ± 1.82, respectively.

**Table 2:** The Measurement Based on The First Observation.

Variable	Fish-based supplement (n = 166)	Nutritious biscuits (n = 14)	Combination (n = 39)	Overall
Age [month]	30.2 ± 12.2	26.1 ± 9.5	28.5 ± 11.4	35.25 (14.60)
Gender				
Female	87 (77.68%)	5 (4.46%)	20 (17.86%)	228 (47%)
Male	79 (73.83%)	9 (8.41%)	19 (17.76%)	255 (52%)
Average Height [cm]	79.47 ± 8.52	76.50 ± 7.30	78.24 ± 8.23	79.06 ± 8.38
Average Weight [kg]	9.69 ± 2.29	8.44 ± 1.21	9.08 ± 2.17	9.50 ± 2.23
BMI [kg/m^2^]	15.27 ± 2.37	14.53 ± 1.91	14.75 ± 2.39	15.13 ± 2.35
Stunting				
Mean HAZ-score	− 3.20 ± 1.01	− 3.62 ± 1.00	− 3.28 ± 1.20	− 3.26 ± 1.06
Normal (HAZ > − 2) [%]	–	–	–	–
Stunting (− 3 < HAZ < − 2) [%]	92 (76.67%)	5 (4.17%)	23 (19.17%)	120 (54.79%)
Severely Stunting (HAZ < − 3) [%]	74 (74.75%)	9 (9.09%)	16 (16.16%)	99 (45.21%)
Wasting				
Mean WHZ-score	− 0.92 ± 1.76	− 1.69 ± 1.38	− 1.38 ± 1.82	− 0.90 ± 1.74
Normal (WHZ > − 2) [%]	124 (78.98%)	8 (2.56%)	25 (12.82%)	157 (71.69%)
Wasting (− 3 < WHZ < − 2) [%]	21 (72.41%)	2 (6.90%)	6 (20.69%)	29 (13.24%)
Severely Wasting (WHZ < − 3) [%]	21 (63.64%)	4 (12.12%)	8 (24.24%)	33 (15.07%)
Number of Public Health Centers	17			

Following the six-month supplementary program from the local government of Serdang Bedagai Regency, the mean height of children in this study noticeably increased by 8.10 ± 5.19 cm (*P*-value  = 2.67e^−19^) to 87.17 ± 8.60. The mean weight of children also significantly increased by 2.65 ± 1.97 kg (*P*-value  = 1.10^e−25^) to 12.15 ± 2.54 (Table 3).

**Table 3:** Measurement after Six-month of Supplementation.

Demographic characteristics	Count
Mean age [month]	37.39 ± 11.89
Mean height [cm]	87.17 ± 8.60
Mean weight [kg]	12.15 ± 2.54

**Figure 3:**
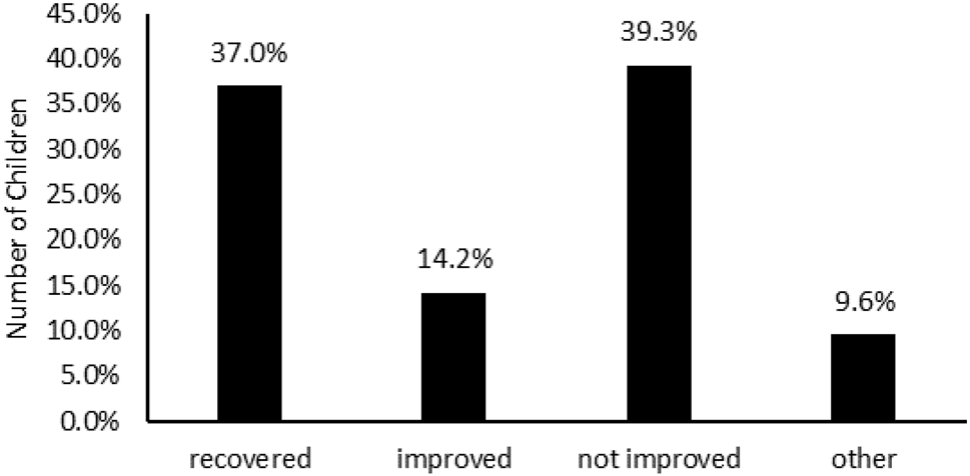
The progress after 6 months supplementary program. The graph indicated that the program gained the recovered rate of 37% and improve the status of children by 14.2%. The improved means that the children with HAZ-score of under − 3 and passed it to − 3 < HAZ < 2.

In addition, Fig. 3 revealed that the proportion of children who had an initial stunted status (HAZ < − 2) and recovered to a normal status (HAZ > − 2) by the final observation period was 37.00% (n = 81), and 14.16% (n = 31) of the children were depicted to improve the status from severely stunted (HAZ > − 3) to moderately stunted (− 3 < HAZ < − 2). Mean HAZ score was also observed to improve significantly with an overall of − 2.29 ± 1.46 or gained about 0.97 ± 1.45 (*P*-value  = 2.40e^−8^) in the span of six months (Table 4). Besides, the mean WHZ-score also obviously improved by 1.00 ± 2.18 (*P*-value  = 2.40e^−8^) to − 0.10 ± 1.91. The number of wasted children also reduced to only 13.24% (n = 29) from previously 28.31% (n = 62).

**Table 4:** Mean Growth Improvement after Six-month Intervention Programs.

Demographic characteristics	Gain	*P*-value
Mean height (sd) [cm]	8.10 ± 5.19	2.67e^−19^*
Mean weight (sd) [kg]	2.65 ± 1.97	1.10e^−25^*
Mean HAZ-score	0.97 ± 1.45	1.74e^−14^*
Mean WHZ-score	1.00 ± 2.18	2.40e^−8^*

*Significant at *P*-value  < 0.05.

Further, Table 5 shows that the children who received supplementation displayed small different mean HAZ scores between categories of supplementation, which were − 2.18 ± 1.49, − 2.92 ± 1.29, and − 2.55 ± 1.31 for fish-based supplement, nutritious biscuits, and combination, respectively. Furthermore, the group that received fish-based supplementation was also, once again, monitored to own the lowest mean WHZ score by 0.05 ± 1.84 and become the only group that passed a positive value (above WHZ-score 0). Children received nutritious biscuits and combination recorded mean WHZ score of − 0.82 ± 1.41 and − 0.48 ± 1.30.

**Table 5:** Measurement after Six-month of Supplementation from Local Government of Serdang Bedagai regency.

Variable	Fish-based supplement (n = 166)	Nutritious biscuits (n = 14)	Combination (n = 39)	Overall
Age [month]	38.13 ± 12.14	32.57 ± 9.00	35.97 ± 11.43	37.39 ± 11.89
Height [cm]	87.92 ± 8.51	82.71 ± 7.74	85.55 ± 8.78	87.17 ± 8.60
Weight [kg]	12.48 ± 2.59	10.38 ± 1.50	11.36 ± 2.22	12.15 ± 2.54
BMI [kg/m^2^]	16.18 ± 2.71	15.28 ± 2.05	15.47 ± 1.75	16.00 ± 2.54
Stunting				
HAZ [cm]	− 2.18 ± 1.49	− 2.92 ± 1.29	− 2.55 ± 1.31	− 2.29 ± 1.46
Normal (HAZ > − 2) [%]	66 (81.25%)	4 (5.00%)	11 (13.75%)	81 (37.00%)
Stunting (− 3 < HAZ < − 2) [%]	58 (77.33%)	2 (2.67%)	15 (20.00%)	75 (34.24%)
Severely Stunting (HAZ < − 3) [%]	42 (67.19%)	8 (12.50%)	13 (20.31%)	63 (28.77%)
Wasting				
WHZ [kg]	0.05 ± 1.84	− 0.82 ± 1.41	− 0.48 ± 1.30	− 0.10 ± 1.91
Normal (WHZ > − 2), [%]	148 (77.90%)	10 (5.26%)	32 (16.84%)	190 (86.76%)
Wasting (− 3 < WHZ < − 2), [%]	9 (75.00%)	1 (8.33%)	2 (16.67%)	12 (5.48%)
Severely Wasting (WHZ < − 3), [%]	9 (52.94%)	13 (17.65%)	5 (29.41%)	17 (7.76%)

Table 6 depicts the growth improvement in height and weight, respectively, among the participants of each supplementary group. The results from both tables indicate that the children’s growth improvement in height and weight was greater in the children who consumed fish-based supplementation, but the difference was not significant (*P*-value = 0.172 and 0.125 for height and weight, respectively). The gender also seemed not to affect to the difference in growth improvement both height and weight for all supplementary groups (*P*-value ≥ 0.05).

**Table 6:** Comparison of The Growth Improvement in Height and Weight between Gender for Each Group of Supplementation from Local Government of Serdang Bedagai Regency.

Groups of Supplementation	Male	Female	*P*-value	Overall
Fish-based supplement				
Height (sd) [cm]	8.16 (± 4.56)	8.71 (± 5.83)	5.03E−01	8.45 (± 5.26)
Weight (sd) [kg]	2.81 (± 2.26)	2.79 (± 2.04)	9.68E−01	2.80 (± 2.14)
Nutritious biscuits				
Height (sd) [cm]	6.94 (± 5.04)	4.88 (± 4.33)	4.57E−01	6.21 (± 4.74)
Weight (sd) [kg]	1.90 (± 1.08)	2.00 (± 1.21)	8.76E−01	1.94 (± 1.08)
Combination				
Height (sd) [cm]	8.88 (± 5.32)	5.82 (± 4.05)	5.00E−02	7.31 (± 4.90)
Weight (sd) [kg]	2.57 (± 0.98)	2.00 (± 1.50)	1.69E−01	2.28 (± 1.29)

*Significant at *P*-value < 0.05.

Further, Table 7 describes the results of the paired t-test to test the significant improvement of mean HAZ-score and mean WHZ-score variables within each category of supplementation. The significant improvement of mean HAZ-score was monitored only among children supplemented by fish-based supplement (*P*-value = 6.59e^−17^) with an improvement of 1.04 ± 1.44. The other two supplementations showed insignificantly different between mean HAZ-score from the initial and final observation (*P*-value > 0.05). A significant improvement in the mean WHZ-score was also recorded by children consuming fish-based supplements (*P*-value = 2.32^e−08^) with an improvement of about 1.04 ± 2.29. Mean WHZ-score improvement (0.83 ± 1.84) also monitored from children obtaining combination supplement (*P*-value = 5.48e^−05^).

**Table 7:** The Improvement Mean of HAZ-score, and WHZ-score after Six-month Intervention Programs (initial vs final observation).

Groups of supplementation	HAZ-score		WHZ-score	
	Improvement	*P*-value	Improvement	*P*-value
Fish-based supplement	1.04 ± 1.44	6.59e^−17^*	1.04 ± 2.29	2.32e^−8^*
Nutritious biscuits	0.73 ± 1.45	0.08	0.87 ± 1.62	0.07
Combination	0.75 ± 1.52	0.75	0.83 ± 1.84	5.48e^−5^*

*Significant at *P*-value < 0.05.

Meanwhile, Table 8 describes the results of one-way ANOVA to test the significant difference in changes of several anthropometric variables within different groups of supplementation. Using a significant level of 5% again, the tests show that there were no significant mean differences in heigh improvement (*P*-value  = 0.172) and weight improvement (*P*-value  = 0.125) within the three groups of supplementation for all participants.

**Table 8:** The comparison of the improvement of mean of height and weight after six-month intervention programs between categories of supplementation.

Variable	Groups of Supplementation			*P*-value
	Fish-based supplement	Nutritious biscuits	Combination	
Height [cm]	8.45 ± 5.27	6.21 ± 4.74	7.31 ± 4.90	0.172
Weight [kg]	2.80 ± 2.14	1.94 ± 1.08	2.28 ± 1.29	0.125

*Significant at *P*-value < 0.05.

**Figure 4:**
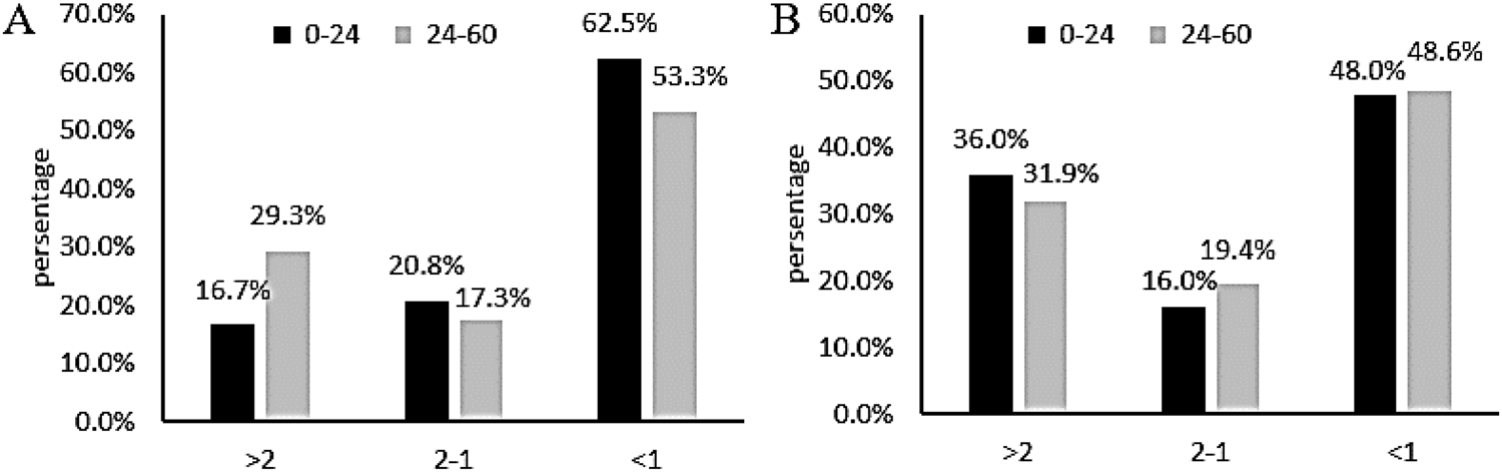
Mean HAZ-score (**A**) & mean WHZ-score (**B**) improvement per age group.

Finally, Fig. 4A indicated that the mean HAZ-score and mean WHZ-score in age group of 0–24 months responded differently. The majority improvement in HAZ-score up to more than 2 was reached by age group of 24–60 months children with about 29.3%, compared to the group of children of 0–24 months with 16.7%. Comparatively, the children aged 0–24 months tended to reach mean HAZ-score improvement at around 1–2 points. Nevertheless, the most of children population underwent mean HAZ-score improvement of under 1 point with 62.5% and 53.3% for 0–24-month and 24–60-month children. In contrast, the highest percentage of mean WHZ-score improvement of more than 2 points was occupied by age group of 0–24 months at 36.0%, while a group of 24–60 months was at 31.9% (Fig. 4B). As mentioned above, the majority of children only improve their WHZ-score by less than 1 for all age groups.

## Discussion

This study provides evidence that the 6-month supplementation program significantly improved the prevalence of stunting in under-5-year-old children in Serdang Bedagai Regency, a newly established Regency in North Sumatra. The one-semester supplementation program initiated by the local government effectively helped the children suffering from stunting to recover, or at least, to improve the condition. The effective improvement was reflected in the improvement of mean HAZ-score and WHZ-score, particularly for children receiving fish-based supplements.

In a study from Jannat et al*.*^12^, the supplementation using yogurt in Dhaka found no significance in the mean HAZ-score or WAZ-score compared to the control. The mean HAZ-score improvement was only 0.13 (*P*-value  = 0.31). Another study by Stephenson et al*.*^13^ in Malawi indicated that 24 weeks of supplementation with cowpea (*Vigna unguiculata*) or common beans (*Phaseolus vulgaris*) showed no improvement in mean HAZ-score, yet the decrease in mean HAZ-score was observed. In contrast, the delivery of ready-to-use supplements in rural Guinea-Bissau showed effectiveness in improving the mean HAZ-score and mean WHZ-score in 3-month delivery of supplements by 1.31 ± 0.79 and 0.28 ± 0.93 in children under 24 months, respectively. In older children up to 59 months, the improvements of mean HAZ-score and mean WHZ-score were 0.83 ± 0.52 and—0.07 ± 0.92, subsequently^14^. Similarly in our study, the improvement of mean HAZ-score and mean WHZ-score of children were found to be 0.97 ± 1.45 and 1.00 ± 2.18 in 6-month supplementation.

The dietary intake in children is important, especially animal-based food. The study by Zaharia et al. indicated that animal-based food has a strong correlation with the HAZ-score and stunting in 6–24 months-old children in Nepal and Bangladesh^15^. Another global literature study from Headey et al.^16^ indicated that the area where fish is included in children’s (age 6–23 months) dietary menu owns a mean stunting prevalence of about 19% (7.6—31.3%). The prevalence was lower than other animal-based diets including eggs only (22.4%) and meal only (24.3%). Animal-source protein including fish also improved HAZ-score among children aged 12–36 months in rural Malawi (*P*-value  = 0.047)^17^. In addition, the study from Marinda et al.^18^ in Zambia indicated that there was a correlation between fish consumption and the stunting prevalence in children aged 6–23 months (*P*-value  = 0.004). They mainly consume small fish from the species *Limnothrissa miodon* that is captured from Lake Tanganyika nearby. Children consuming fish were more frequently discovered to be within the normal range of HAZ scores than their counterparts. Yet, the study found that fish consumption has no significant association with WAZ or WHZ scores.

In contrast, the finding in this study is not linear with the finding from the study conducted in Kenya by Konyole et al.^19^. The study indicated that the fortification of small cyprinid fish called *Rastrineobola argentea* into their food did not promote growth and body composition compared with the control. In another study from Cambodia by Skau et al.^20^, the supplementation conducted using locally available fish (*Esomus longimanus* and *Paralaubuca typus*) found that the addition of fish in fortified food resulted similarly with control. The proportion of the fortified fish in the functional food and the species of the fish probably affect the insufficiently.

In our study, the local government promoted fish-based supplement obtained from local snakehead fish (*Channa striata*) that is abundantly available. As one of the fish species with the highest protein contents, *C. striata* has become one the most promising protein sources to be used for the prevention and treatment of stunting. This species has been reported to have a high content of protein, complete essential amino acids, fatty acids, and other essential nutrients, which are crucial for children’s growth. In addition, low collagen and high albumin protein contents in *C. striata* meat also provide high digestibility in babies, children, and elderly people as well as people who are recovering from illness, respectively^21^. A study by Kundarwati et al. reported that protein intake, zinc and iron significantly correlated with the incidence of stunting in 60 toddlers aged 1–3 years^22^. In this regard, 100 mL of *C. striata* extract was reported to contain high albumin (2.17 ± 0.14 g), zinc (3.34 ± 0.8 mg), and iron (0.20 ± 0.09 mg), which is sufficient to provide albumin for highly demanded such as hypoalbuminemia and growing children. The Zn content in the C. striata extract is also adequate to fulfil the daily requirements of diet intake for toddlers^23,24^^.^.

The other advantage of using locally provided food is that it reduces the cost of supplementation programs. Although the national government provided nutritious biscuits from the National Supplementary Feeding Program, the availability is limited. The national government also focuses on delivering the biscuits only for children with underweight, thus children with normal weight but stunted cannot access the biscuits supplement. In addition, utilizing fish-based supplements seems to be more promising than biscuits since the improvements in mean HAZ-score and WHZ-score were higher. Although the nutritious biscuit also contained complete nutrients, such as 450 cal, 14 g of fat, 9 g of protein, 71 g of carbohydrates, 10 vitamins (vitamins A, B1, B2, B3, B6, B9, B12, D, E and K) and 7 minerals (iron, zinc, phosphor, selenium, dan calcium)^4^, it effects on mean HAZ-score improvement was not significant It might be explained by several factors such as the absence of appetite and immunostimulant compounds, as well as the lower digestibility of the nutrient in the group.

Another important feature of supplements for stunting is improving the children’s appetite, wherein stunted children often have poor appetite, which may limit their response to nutritional interventions^24^. The *C. xanthorrhiza* and *P. niruri* contents in the fish-based supplement also probably supported the positive result of the supplementary program from the local government of Serdang Bedagai. *C. xanthorrhiza* and *P. niruri* are two herbals that possess appetite stimulant activity^25,26^. *C. xanthorrhiza* has been extensively utilized in Indonesia as a medicinal and nutritional plant, which is traditionally used to treat several ailments, including lack of appetite. Major active compounds of *C. xanthorrhiza* are terpenoids, curcuminoids, and other phenolic compounds, which have been correlated with its ethnopharmacological properties such as antioxidant, antimicrobial, anti-inflammatory, anticancer and antitumor, antidiabetic, and hepatoprotective^25^. On the other hand, the active phytochemicals compounds identified from *P. niruri* were flavonoids, alkaloids, terpenoids, lignans, polyphenols, tannins, coumarins, and saponins. Those active compounds have been reported to be effective against Hepatitis B and other viral infections^27^.

Finally, the result of this research became an insight for local health policymakers to optimize the food that is abundantly available, especially fish-sourced food or other animal-sourced food. The evidence can also be suggestion for the national government to establish a national initiative plan to reduce stunting prevalence in Indonesia^28,29^. By optimizing the local resources and biodiversity, the rural area could independently eradicate stunting or malnutrition. Some countries including Zambia and Kenya could utilize locally available food sources to supplement the children with stunting and malnutrition^18,30^. More evidence also revealed that the children living within the area of fish-farming households in Malawi were less prevalent of underweight and malnutrition^31^.

## Conclusion

The government of Serdang Bedagai regency has implemented stunting reduction programs by delivering food supplements, fish-based supplements, and nutritious biscuits to reduce the prevalence of stunting in the area. As an effort to monitor and evaluate the program, we provided evidence that the program could effectively reduce the prevalence of stunting in children below 5-years, especially among children who received locally produced fish-based supplements. Due to the success of the supplementary program, the government could continue the program until the stunting is eradicated from the area. The improvement of HAZ scores and WLS scores become evidences that further supports the continuity of the program. The use of locally sourced protein in this program can also be applied in other developing areas that have similar stunting and malnutrition problems.

## Abbreviations

HAZ-score: Height-for-age Z-score
WHZ-score: Weight-for-height Z-score
WHO: World Health Organization
SFP: Supplementary Feeding Program (program from national government)
BPS: Statistics Indonesia: Indonesian Central Bureau of Statistics
DBMS: Database Management System
BDSRC: Data Science Research Center (Bina Nusantara University)
StuntingDB: Database management system (DBMS) to manage stunted children’s data
N: Number of samples
BMI: Body Mass Index
